# Development of a Three-Dimensional Geometric Model of Multi-Structured Woven Fabrics Using Spun Yarns for Theoretical Air Permeability Prediction

**DOI:** 10.3390/ma19051045

**Published:** 2026-03-09

**Authors:** Theeradech Songart, Wasit Chaikumming, Keartisak Sriprateep

**Affiliations:** Manufacturing and Materials Research Unit (MMR), Department of Manufacturing Engineering, Faculty of Engineering, Mahasarakham University, Mahasarakham 44150, Thailand; teeradet.s@msu.ac.th (T.S.); wasit.ckm@gmail.com (W.C.)

**Keywords:** air permeability, woven fabric, filament assembly model, CAD, CFD, textile simulation

## Abstract

This study presents the development of a three-dimensional (3D) filament assembly model for predicting the air permeability of woven fabrics composed of spun yarns. To address the limitations of conventional single-line yarn models, the proposed framework incorporates fiber-level geometric representations using non-uniform rational B-splines (NURBS) and simulates multiple weave patterns—including plain, basket, twill, and rib—under various set density configurations. Each yarn was modeled with accurate filament distribution and cross-sectional layering, enabling the construction of realistic unit-cell-based CAD geometries. Computational fluid dynamics (CFD) simulations were performed using the k-ε turbulence model in SolidWorks Flow Simulation and validated against experimental measurements conducted under ISO 9237:1995 conditions. The filament assembly model achieved high predictive accuracy, exhibiting a lower of percentage prediction errors than the single-line yarn path model, thereby more effectively capturing airflow behavior through inter-yarn and intra-yarn pores. These findings highlight the capability of integrated CAD/CFD methodologies for virtual prototyping of breathable textiles and provide a robust foundation for high-precision performance prediction in functional and technical fabric design.

## 1. Introduction

Air permeability is defined as the rate at which air flows perpendicularly through a unit area of fabric under a specified pressure gradient over a given time period. This property is one of the most critical factors influencing fabric comfort. A precise assessment of the air permeability of textile fabrics during the design stage is essential, as it can significantly reduce time and costs in the development of new fabric structures. Additionally, such assessment ensures the necessary functionality of textile products [1]. The primary factors affecting the air permeability of fabrics include porosity, pore characteristics, fluid properties passing through the fabric, and environmental factors. Yarn diameter, yarn type, and warp/weft densities also influence the porosity of the fabric. Therefore, controlling the permeability performance of a fabric involves managing its pore properties, fluid properties, and environmental factors. Burleigh et al. [2] first introduced the concept of “effective porosity,” a key determinant of flow, which can be visualized in three components: (a) intrafiber porosity the void space within the fiber walls; (b) interfiber porosity the void space between fibers in the yarn; and (c) inter-yarn porosity the void volume created by the interstices between the yarns.

Historically, many studies have focused on predicting the air permeability of woven and knitted fabrics based on their structural parameters. Backer [3], for instance, analyzed the relationship among four intersecting yarn types, simplifying the complex fabric structure and airflow by modeling yarns as flexible, inextensible, circular cylinders. Penner et al. [4] visually investigated flow through fabrics, treating pores as orifices. Their study examined the influence of weave structure on fluid flow within a Reynolds number range of 10 to 400. They found that the effective pore area was not constant and decreased with increasing Reynolds number. Similarly, Kulichenko and Langenhove [5] predicted the permeability of woven fabrics using Poiseuille’s formula for laminar flow and Darcy’s law for both laminar and turbulent flow, incorporating corrections proposed in earlier studies. Lu et al. [6] numerically analyzed fluid flow through basic weaves of monofilament filter cloth using FLUENT software. Their results highlighted the significant influence of pore characteristics on flow patterns within interstices and downstream regions. Experimental studies have further explored the effects of structural parameters on air permeability. For example, Gooijer et al. [7] developed a model to predict the flow resistance of multifilament fabrics. While effective for general flow resistance modeling, their method was unsuitable for deformed fabrics. Xu and Wang [8] validated a model based on the Hagen Poiseuille equation, demonstrating strong agreement between predicted and experimental data. Zupin et al. [9] examined the air permeability of single-layer woven fabrics using porosity parameters. They identified three predictors—hydraulic diameter of pores, pore count, and total porosity—and used statistical methods to establish a prediction model. Their approach effectively described the relationship between fabric structure and permeability using staple yarn fabrics. Multiple linear regression was applied to derive a predictive equation for permeability [10,11,12,13].

Over the last two decades, computer-aided design (CAD) and computational fluid dynamics (CFD) systems have advanced significantly, enabling the development of 3D models for woven and knitted fabric structures. Geometric modeling of yarn structures now often incorporates single-line yarn paths [14,15,16,17,18] or filament assemblies twisted in CAD environments [19,20,21,22,23,24]. Sriprateep [25] successfully employed CAD/CAE models with filament assemblies to predict yarn stress–strain curves. In these applications, airflow through the fabric structure is a critical consideration. Predicting air permeability through CFD techniques is increasingly recognized as a valuable asset in the engineering design of textile structures. Numerous studies have leveraged CFD to predict air permeability in knitted [1,26,27,28,29,30] and woven fabrics [31,32,33,34,35,36,37]. Tung et al. [31] numerically examined how woven structures influence fluid flow through multifilament woven filter cloths using FLUENT software. Their findings revealed that pore construction significantly affects flow patterns, with plain weaves offering the highest resistance and satin weaves the lowest under identical thread counts. Kulichenko [32] modeled woven fabric geometry with virtual imaging, highlighting the correlation between 3D pore morphology and construction parameters. Wang et al. [33] derived equations to calculate constricted open areas and discharge coefficients for plain-woven monofilament fabrics. They observed a logarithmic relationship between the discharge coefficient and Reynolds number through numerical simulations. Angelova et al. [34] created virtual models of single-layer woven fabrics, incorporating pore size, shape, and distribution into CFD software to evaluate permeability. Their simulations aligned well with experimental results. Similarly, Angelova et al. [35] modeled the through-thickness air permeability of woven structures using CFD and turbulence models (k-ɛ, k-ω, and Reynolds stress models). Kyosov et al. [36] extended these methods to study air permeability in textile layer ensembles, demonstrating the applicability of CFD for further investigations of multilayer systems. Recently, Xie et al. [37] developed a three-dimensional geometric model and performed numerical simulations of airflow resistance and of the nonlinearity coefficient (NLF) using the finite volume method, with experimental permeability tests confirming the model’s reliability through errors of less than 10%.

In addition to advancing numerical prediction accuracy, the integration of filament-level geometric modeling with CFD offers substantial potential for practical applications in textile engineering and product development. By enabling virtual evaluation of fabric breathability at the design stage, such CAD/CFD frameworks can significantly reduce reliance on extensive physical prototyping and experimental trials. This capability is particularly important for functional and technical textiles, including filtration media [33], sportswear [38], and auto-racing suits [39], where precise control of air permeability is essential to performance and user comfort. Moreover, embedding CAD/CFD-based permeability prediction into digital textile design environments can enhance predictive and performance-driven design workflows. Despite these advantages, several challenges remain that motivate further investigation, including the incorporation of yarn hairiness and surface irregularities to better represent realistic airflow resistance [40], the consideration of dynamic pore deformation under varying pressure conditions, and the adoption of advanced turbulence modeling and mesh adaptivity near yarn interfaces. Addressing these aspects would further extend the applicability of filament-level CAD/CFD models toward multilayer fabrics, coated textiles, and other complex industrial fabric systems.

Despite these advancements, limited research exists on applying CFD to model and simulate air permeability in plain, twill woven fabrics, filling rib and warp rib. This study addresses this gap by developing a 3D filament assembly model to predict the air permeability of woven fabric structures. Woven samples with varying patterns and warp/weft densities were produced and simulated, ranging from open to tight structures. Geometric modeling of the fabrics was performed in SolidWorks, and CFD simulations were conducted using SolidWorks Flow Simulation 2021 software. Numerical results were compared against experimental measurements of air permeability for the woven samples studied.

## 2. Materials and Computational Methodology

### 2.1. Materials

This study adopts reference data reported by Zupin et al. [9], including the woven fabric structural types, their corresponding geometric parameters, and the experimental air permeability results. Six types of fabric structures were selected: plain weave (PL), basket weave (BW), twill 1/3 (T1/3), twill 2/2 (T2/2), filling rib (R4/2) and warp rib (R2/4). In each fabric structure, the warp densities selected were 22 and 29.3 ends/cm, while the weft densities were 15 and 20 picks/cm. Consequently, the samples were divided into four groups based on density combinations: 22/15, 22/20, 29.3/15, and 29.3/20 yarns/cm. It is noteworthy that the pore sizes of fabric structures varied within these groups. The warp and weft yarns used in all fabric samples were cotton with an identical linear density of 17.2 tex. All fabric samples were woven using a Jettis NFB 190 weaving loom (Jettis Textile Machinery Ltd., Ljubljana, Slovenia) under controlled laboratory conditions. Fabric thickness and mass per square meter were measured following standardized procedures. Set and construction parameters for 24 woven fabric samples are shown in Table 1. The optically determined warp and weft yarn diameters were 0.263 mm and 0.282 mm, respectively, across all samples. Air permeability testing was conducted using an Air Permeability Tester III Fx 3300 LABOTESTER in compliance with ISO 9237:1995(E) [41]. The test area was 20 cm^2^ under a pressure difference of 200 Pa. Ten repeated measurements were performed for each fabric sample to ensure statistical reliability.

### 2.2. CAD Modeling of Woven Fabrics

**Figure 1:**
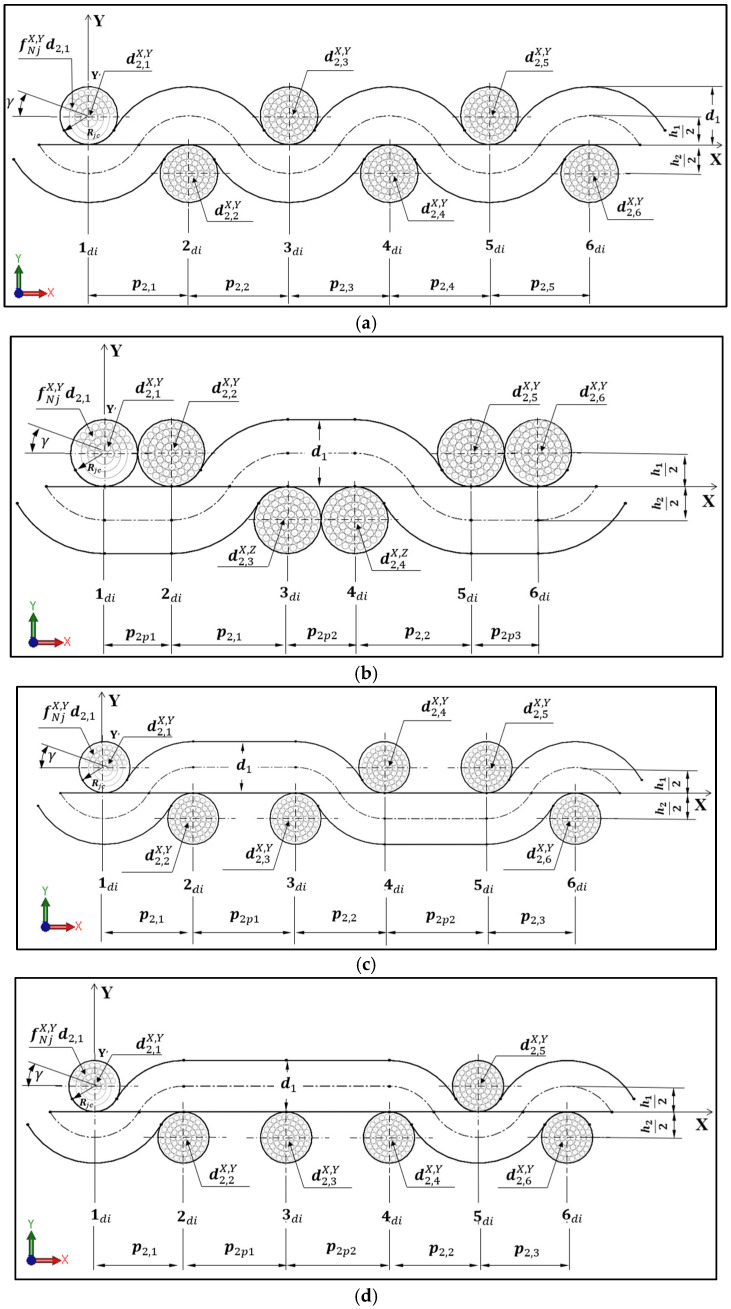
Geometric model of (**a**) 1 × 1 plain weave, (**b**) 2 × 2 basket weave, and (**c**,**d**) 2 × 2 and 3 × 1 twill weave, respectively.

In this study, Peirce’s classical model is employed solely as a preliminary topological and kinematic reference for defining yarn interlacement and centerline trajectories, while the pore geometry governing airflow is entirely determined by a filament-resolved solid model and is therefore independent of Peirce’s idealized yarn assumptions. The coordinate systems and geometrical models of yarn in plain, basket, and twill weave fabric structures are illustrated in Figure 1. The position of the central fabric structure is defined by the Cartesian coordinates (X, Y), where the center of the woven fabric structure is located at (X = 0, Y = 0). This model represents weave interlacements, where the yarns are considered inextensible and flexible. The yarns are assumed to have a circular cross-section and are composed of both straight and curved segments. In the case of the 4 × 2 filling rib fabric type, coordinate systems and geometrical models of the form 1 × 1 PL in XY plane and 2 × 2 basket in YZ plane can be used. In contrast, in the case of the 2 × 4 warp rib fabric type, coordinate systems and geometrical models of the form 2 × 2 basket in XY plane and 1 × 1 PL in YZ plane can be used.

The geometric parameters of fabric structures input into the model and the major steps for constructing a 3D model of a woven fabric structure were the same as in the study by Patumchat and Sriprateep [19]. The construction of the three-dimensional (3D) fabric model involved three primary steps. Step 1: The woven fabric structure was initially described in 2D based on Peirce’s model and the yarn cross-section geometry. Step 2: A modeling method was developed to represent the warp and weft yarns for the 3D model. Step 3: The final stage involved creating a solid CAD model of the fabric structure. The geometric parameters input into the single-line yarn path model included warp and weft yarn diameters, warp and weft yarn spacing, warp and weft half crimp wave heights, sinking or floating warp/weft spacing, and warp and weft crimp angles. These parameters were determined using the set density values provided in Table 1, and the coordinate system of woven fabrics is illustrated in Figure 1. The filament assembly model required additional geometric parameters, including the filament diameter, the number of filaments within the yarn cross-section, the number of layers, and the yarn twist angle.

**Figure 2:**
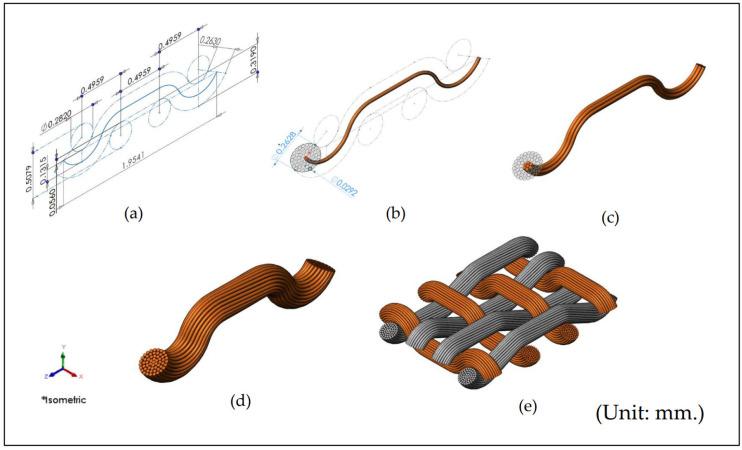
CAD model of unit cells fiber assembly of the 22/15 of 2 × 2 twill weave: (**a**) definition of the yarn centerline geometry; (**b**) creation of the filament bundle cross-section; (**c**) sweeping of filaments along the yarn path; (**d**) completed filament-based yarn model; (**e**) assembly of yarns to generate the woven fabric structure used in the CFD simulation. The symbol labeled Isometric indicates that the CAD model is being displayed in a standard 3D isometric orientation, allowing all three spatial dimensions of the object to be viewed simultaneously for better visualization and analysis.

The geometric structures of six woven fabric types were simulated at four different set densities in the warp and weft directions using SolidWorks 2021 software. A total of 24 unique fabric structures, varying in weave patterns and set densities, were modeled. Figure 2 illustrates the example steps involved in designing a 3D filament assembly model for 1-unit cell of a 2 × 2 twill weave fabric with set densities of 22/15. The design process for each model began with creating a two-dimensional sketch of the woven fabric structure’s axis on a plane, utilizing non-uniform rational basis spline (NURBS) curves. The curve’s shape was defined based on the coordinate system of the fabric and the warp/weft densities using the set fabric density values. For instance, in the case of the 2 × 2 twill weave fabric with 22/15 set densities, the warp and weft yarn spacings were 1.682 mm and 0.603 mm, respectively, while the warp and weft yarn diameters measured 0.263 mm and 0.282 mm, respectively. The second step involved sketching the warp and weft profiles in a plane perpendicular to the axis sketch. By applying the projection operation to the first sketch, a three-dimensional axis for the warp and weft was generated. Subsequently, a cross-sectional sketch of the yarn, composed of individual fibers, was created. The final warp/weft shapes were achieved by performing a swept boss or base operation on the profiles generated in the previous steps.

### 2.3. CFD Analysis

#### 2.3.1. Equations Describing the Fluid Flow

Computational fluid dynamics (CFD) simulations in this study were performed using SolidWorks Flow Simulation, which solves the governing conservation equations for mass, momentum, and energy on three-dimensional computational domains with user-defined boundary conditions. Rather than presenting the full instantaneous Navier–Stokes formulation, the analysis focuses on identifying the relevant flow regime and implementing an appropriate numerical framework for air permeability evaluation through knitted fabrics. The airflow was driven by a prescribed pressure difference across the fabric thickness, and the resulting velocity field predominantly developed in the direction normal to the fabric plane. Under these conditions, the flow behavior can be reasonably approximated as quasi–one-dimensional at the macroscopic scale, while the local velocity variations are resolved by the three-dimensional filament-level geometry.

**Figure 3:**
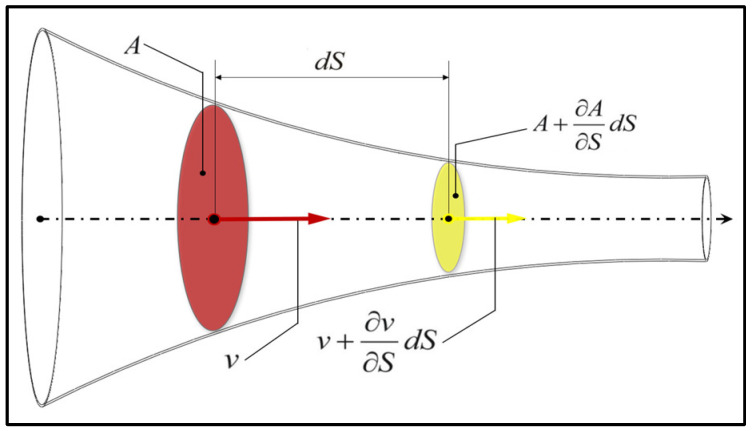
Scheme of fluid flow inside a tube with a variable cross-section.

Let us consider the volume of fluid entering and exiting from the volume reduced by cross-sections A and $A+\frac{\partial A}{\partial S}dS$ in the time dt that is shown in Figure 3. The dS is the distance from cross-section A to cross-section $A+\frac{\partial A}{\partial S}dS$. The mass of the fluid flowing through section A at a velocity v in time dt is ρvAdt, while the mass of the fluid flowing out at a velocity $v+\frac{\partial V}{\partial S}dS$ through section $A+\frac{\partial A}{\partial S}dS$ at the same time is $\left(\rho +\frac{\partial \rho }{\partial S}dS\right)\left(A+\frac{\partial A}{\partial S}dS\right)\left(v+\frac{\partial v}{\partial S}dS\right)dt$. Mass conservation across an infinitesimal control volume along the flow direction is expressed as

(1) $$\rho Avdt-\left(\rho +\frac{\partial \rho }{\partial S}dS\right)\left(A+\frac{\partial A}{\partial S}dS\right)\left(v+\frac{\partial v}{\partial S}dS\right)dt=\frac{\partial \rho }{\partial t}dtAds$$

where ρ is the air density, v is the local velocity, A is the effective flow cross-sectional area, S denotes the spatial coordinate along the flow direction, and t represents time. This formulation describes the net mass balance between inflow and outflow through adjacent cross-sections and forms the basis for permeability evaluation under steady-state conditions. The flow regime was assessed using the Reynolds number defined by the characteristic pore-scale length and the mean air velocity. For all simulated cases, the Reynolds numbers were well below the critical threshold for turbulence, confirming that airflow through the woven fabrics remained laminar. Although SolidWorks Flow Simulation is based on a RANS solver framework incorporating the standard k–ε turbulence model, the flow regime in the present study was confirmed to be laminar based on pore-scale Reynolds number analysis (Re ≈ 2.4–76.9). Under these conditions, no additional turbulent viscosity contribution was generated, and the airflow behavior was governed by viscous laminar transport without low-Reynolds-number turbulence corrections. Instead, the solver framework ensures numerical robustness while the airflow behavior is governed by laminar flow through filament-resolved pore structures.

#### 2.3.2. Boundary Conditions and Numerical Setup of Airflow Simulations

Computational fluid dynamics (CFD) simulations were performed using SOLIDWORKS Flow Simulation 2021, which solves the three-dimensional Reynolds-Averaged Navier–Stokes (RANS) equations via the finite volume method (FVM) for incompressible Newtonian fluids.

I. *Computational Domain and Geometric Representation*—The computational domain consisted of a rectangular flow channel with a total development length of 8 mm, defined as 4 mm upstream and 4 mm downstream of the fabric mid-plane. Unlike conventional configurations where fabric thickness contributes to total channel height, the measured fabric thickness (0.439–0.620 mm; Table 1) was treated as an internal solid obstruction and was not added to the external flow-development length. The lateral dimensions corresponded exactly to the periodic unit-cell dimensions determined by warp and weft spacing, and periodic boundary conditions were imposed to emulate infinite fabric repetition.

II. *Boundary Conditions and Physical Assumptions*—The airflow configuration reproduced a pressure-driven ISO 9237 [41] permeability test with the following conditions:

- Inlet: Pressure inlet, ΔP = 200 Pa;
- Outlet: Environmental static pressure (0 gauge);
- Lateral faces: Periodic boundary condition;
- Yarn surfaces: Impermeable no-slip wall;
- Fluid: Incompressible air at 298 K;
- Gravity: Neglected;
- Flow regime: Steady-state.

**Figure 4:**
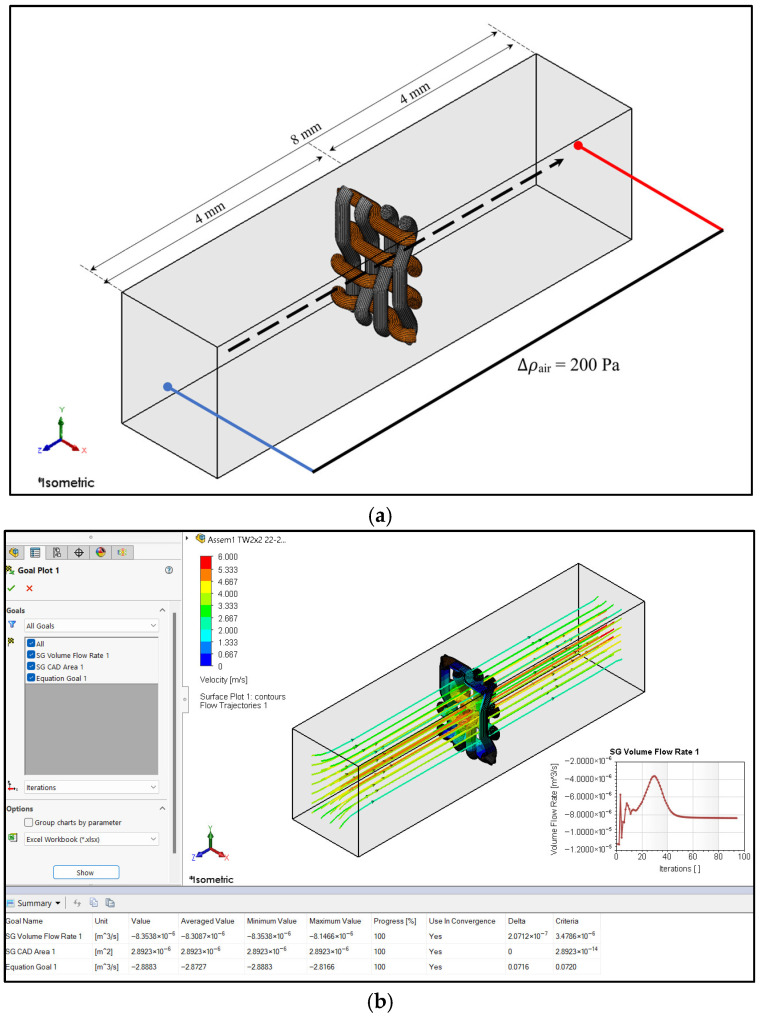
Example of computational method for 2 × 2 twill weave with 22/15 set density: (**a**) simulation conditions and (**b**) air velocity distributions for calculating the air permeability. The symbol labeled Isometric indicates that the CAD model is being displayed in a standard 3D isometric orientation, allowing all three spatial dimensions of the object to be viewed simultaneously for better visualization and analysis.

No empirical calibration parameters were introduced; air permeability emerged solely from the applied pressure difference and measured yarn geometry. Figure 4a shows the computational cell containing one 2 × 2 twill unit cell (22/15 set density), while Figure 4b illustrates the velocity distribution obtained from the simulation. The unit-cell approach significantly reduced computational time.

III. *Flow Regime Assessment*—Pore-scale Reynolds numbers were estimated using the effective hydraulic gap derived from measured yarn diameters, set densities, fabric thickness, and crimp geometry. For velocities corresponding to the 200 Pa pressure drop (approximately 0.5–6 m·s^−1^ within pore constrictions), Reynolds numbers ranged from Re ≈ 2.4 to 76.9 across all cases. This range indicates laminar to weakly inertial porous flow dominated by viscous resistance. Under these conditions, no additional turbulent viscosity contribution was generated.

The applied pressure difference (200 Pa) is several orders of magnitude lower than the mechanical stiffness of cotton yarns; therefore, filament deformation and contact variation were neglected, and yarn geometry was treated as rigid without fluid–structure interaction.

IV. *Numerical Discretization and Mesh Strategy*—The computational domain was discretized using the Cartesian structured mesh of SOLIDWORKS Flow Simulation 2021 (Mesh → Global Mesh), generating orthogonal hexahedral control volumes comprising fluid and interface (partial) cells. Automatic local refinement was applied near high-curvature regions, narrow inter-yarn gaps, intra-yarn voids, and yarn–air interfaces to accurately resolve velocity and pressure gradients within pore constrictions. For a representative case (2 × 2 twill, 22/15 set density; Figure 4), the mesh statistics reported under Mesh → Information indicated approximately 5.5 × 10^4^ Total Cells, including ~4.0 × 10^4^ Fluid Cells and ~1.5 × 10^4^ Partial Cells. A mesh independence assessment confirmed that the variation in predicted air permeability between medium and refined meshes was below 2%, demonstrating mesh-converged solutions.

V. *Mesh Convergence and Numerical Stability*—A mesh independence study using coarse, medium, and fine grids showed that predicted air permeability varied by less than 2% between medium and fine meshes, confirming grid convergence. Convergence criteria included stabilization of pressure drop, mass flow consistency, and residual reduction thresholds. The medium mesh was selected as a balance between accuracy and computational cost.

## 3. Results and Discussion

### 3.1. CAD Model

**Figure 5:**
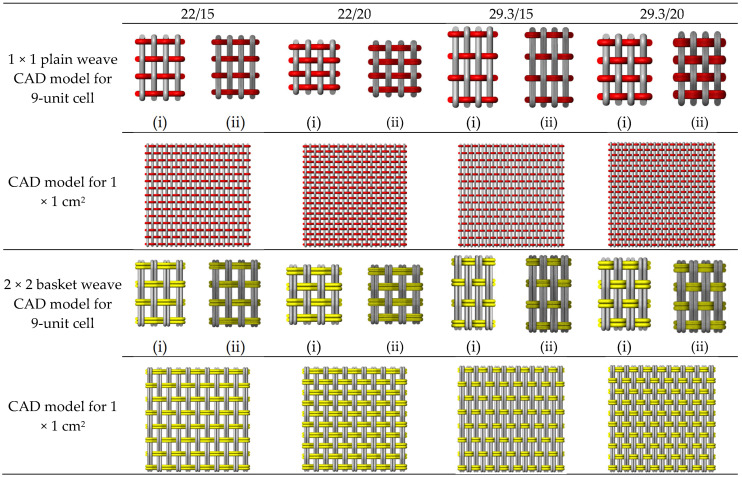

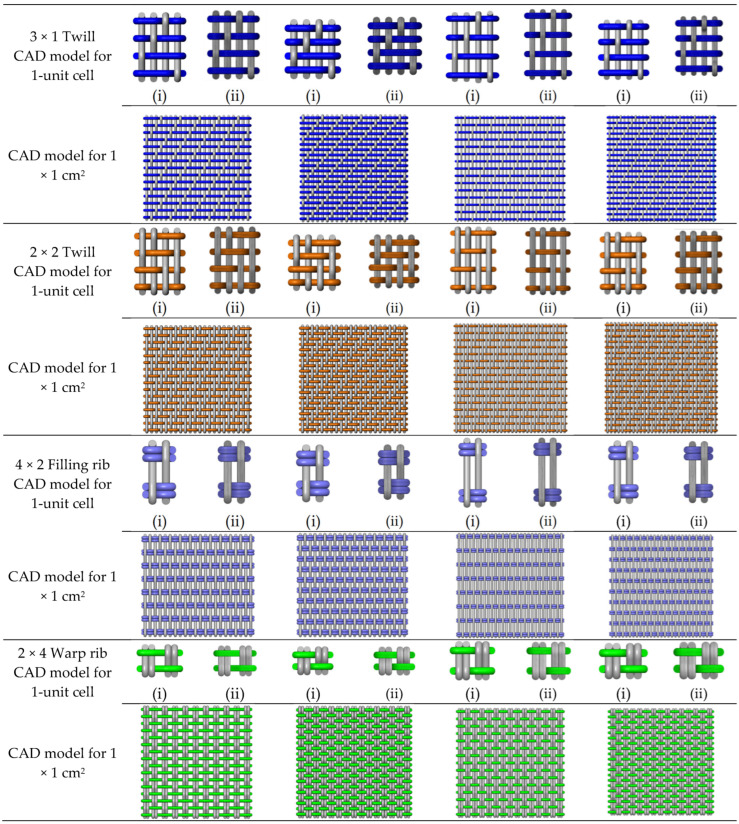
CAD model (top view) of unit cells and 1 × 1 cm^2^ of the surfaces of fabric samples: (i) single-line yarn path and (ii) filament assembly model.

The main steps outlined in Section 2.2 provided the foundation for developing our 3D model of a woven fabric structure. Figure 1 references the geometrical parameters used for input into computer-aided modeling, which were derived from the set densities listed in Table 1. In the CAD model, two approaches, the single-line yarn path and the filament assembly model, were analyzed for each fabric structure to predict air permeability. As a result, the CAD models comprised 48 distinct patterns. For example, in the case of the 1 × 1 plain weave fabric, the warp/weft set density was 22/15 per millimeter (mm), and the fabric thickness was 0.439 mm. For the single-line yarn path, the warp and weft yarn diameters were 0.263 mm and 0.282 mm, respectively. The yarn spacing at the crossover point of the warp and weft was 0.4959 mm, while the half-crimp wave height was 0.319 mm. Additional parameters for the filament assembly model included a filament diameter of 0.0292 mm. The yarn cross-section contained five layers, with 56 filaments per yarn. Figure 5 illustrates the top views of unit cells (1 × 1 cm^2^) for four different set densities of each fabric structure. A total of 48 samples, comprising six weave types, four set densities, and two CAD-based modeling approaches, were subjected to analysis. The warp and weft densities were observed to significantly influence the shapes of the rectangular pores. Increased warp and weft densities within the 1 × 1 cm^2^ area resulted in reduced pore sizes, which will affect air permeability.

### 3.2. CFD Model

**Figure 6:**
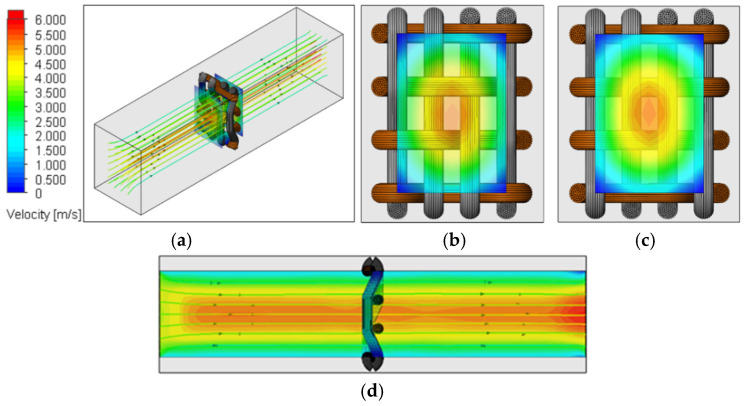
(**a**) The air velocity distributions were obtained for a one-unit cell of the 2 × 2 twill fabric and modeled using the filament assembly approach (**b**) positioned directly above and (**c**) directly below, and (**d**) visualization of the shape of the airflow lines.

Based on the CFD analysis for airflow simulations described in Section 2.3, air permeability was simulated using the single-line yarn path and filament assembly models. An example of the simulation results for the filament assembly model for the velocity field in a 2 × 2 twill weave fabric structure is shown in Figure 6. These results illustrate the air velocity distributions for one unit cell, including airflow directly above and below the fabric, as well as a visualization of the airflow lines. In these simulations, airflow moved from the inlet (left side of Figure 6) to the outlet (right side of Figure 6) due to the pressure difference across the sample. As the airflow encountered the fabric, it deviated toward the pore locations to pass through, resulting in increased velocity. After passing through the fabric, the airflow tended to stabilize.

**Figure 7:**
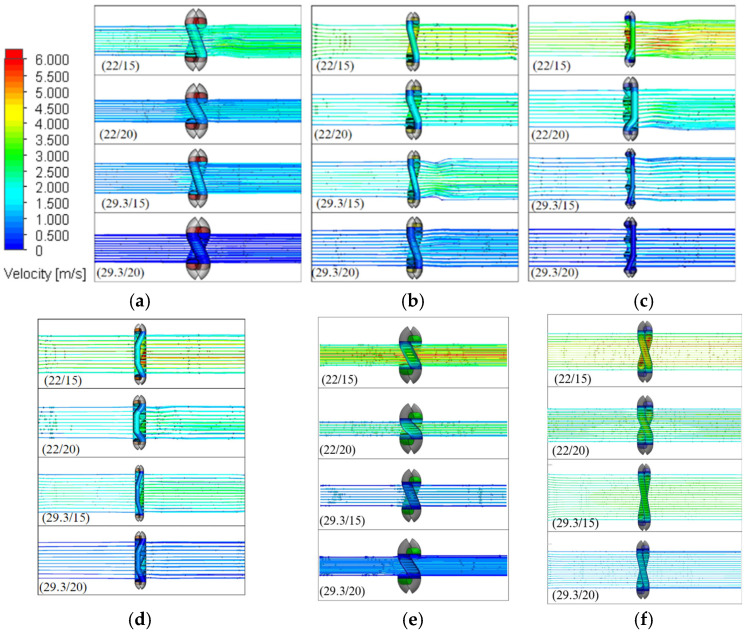
Velocity counters with single line of yarn model: (**a**) 1 × 1 PW, (**b**) 2 × 2 BW, (**c**) T 1 × 3, (**d**) T 2 × 2, (**e**) 4 × 2 filling rib and (**f**) 2 × 4 warp rib.

**Figure 8:**
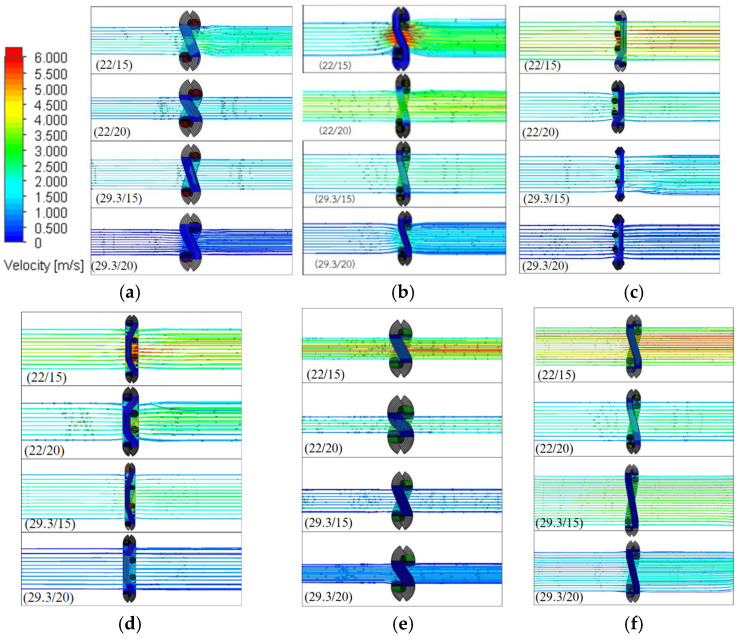
Velocity counters with filament assembly model: (**a**) 1 × 1 PW, (**b**) 2 × 2 BW, (**c**) T 1 × 3, (**d**) T 2 × 2, (**e**) 4 × 2 filling rib and (**f**) 2 × 4 warp rib.

Air permeability refers to the rate at which air passes perpendicularly through a unit area of fabric under a specific pressure gradient over a given time period. The output of air permeability is expressed as the flow rate of air through the fabric (Q). The air permeability (R) was calculated using the equation R = Q/A_t_, where A_t_ represents the tested fabric area. For the CFD analysis, the k-ε turbulence model, which belongs to the Eddy Viscosity Models (EVMs) group, was employed. The simulation results for the velocity field of both the single-line yarn path and filament assembly models are summarized in Figure 7 and Figure 8, respectively. The tested fabric area for each set density and fabric type was calculated based on the dimensions of each unit cell. Increasing the set density reduced pore size, which in turn decreased the air permeability.

Figure 6, Figure 7 and Figure 8 compare the air velocity distributions above and below the fabric surfaces for different weave structures. Above the fabric surface, the airflow accelerates and streamlines concentrate toward pore openings, indicating preferential flow entry through inter-yarn gaps. This concentration is more localized for plain weaves due to their higher interlacement density and smaller effective pore openings, whereas twill and rib weaves exhibit broader high-velocity regions above the fabric surface. Below the fabric surface, the airflow emerges as localized high-velocity jets followed by gradual velocity reduction and redistribution. Plain weaves show shorter jet penetration and faster dissipation, while twill and rib structures display more persistent downstream velocity patterns, consistent with their lower flow resistance and higher air permeability.

Figure 7 and Figure 8 demonstrate a distinct difference in velocity contours between the two geometric models. The single-line yarn model produces highly concentrated jet-like flow through inter-yarn pores, with localized velocity peaks at pore entrances and pronounced downstream jet cores. In contrast, the filament assembly model exhibits a more spatially distributed velocity field, with reduced peak magnitudes and smoother downstream recovery. Partial flow redistribution is observed within the filament-resolved structure, resulting in less concentrated velocity gradients. Overall, the filament assembly model predicts a more uniform airflow distribution across the fabric thickness compared to the simplified yarn representation.

**Figure 9:**
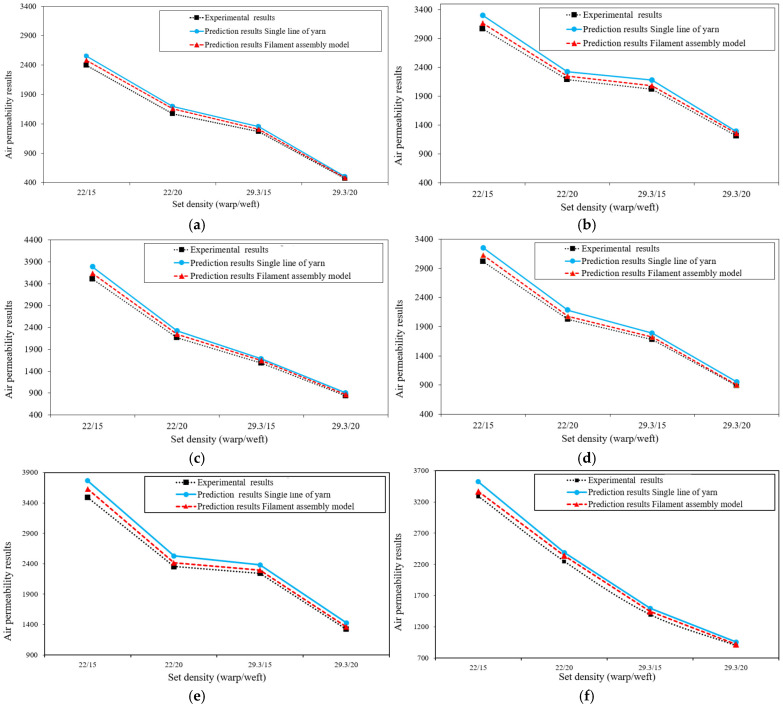
Comparison of air permeability predictions from the single-line yarn and filament assembly models with experimental results reported by Zupin et al. [9] for (**a**) 1 × 1 plain weave, (**b**) 2 × 2 basket weave, (**c**) 1 × 3 twill, (**d**) 2 × 2 twill, (**e**) 4 × 2 filling rib, and (**f**) 2 × 4 warp rib.

The numerical and experimental results are summarized in Table 2. Also, Figure 9 compares the air permeability predictions from the single-line yarn path and filament assembly models with experimental results. The simulation results demonstrated excellent agreement with the experimental data, with the filament assembly model yielding higher accuracy than the single-line yarn path model. Furthermore, the results confirmed that increasing the set density decreases air permeability due to the reduction in pore size. Using the actual structural parameters of each weave fabric yielded accurate simulation geometries. Consequently, CAD/CFD techniques can be applied more precisely to predict the air permeability of fabrics.

### 3.3. Statistical Assessment of Air Permeability Prediction

Applying statistical principles to support the evaluation of predictive models is widely practiced across engineering disciplines [42,43,44]. In the present study, statistical indicators are provided only as supplementary quantitative evidence to confirm the trends already illustrated in Figure 9. Table 3 summarizes the percentage error (% Error) between numerical predictions and experimental results for both geometric modeling approaches. The single-line yarn model shows percentage errors ranging from 6.09% to 7.92%, with a mean value of 7.14% (Standard Deviation (SD) = 0.67). In contrast, the filament assembly model yields lower errors, ranging from 2.03% to 5.35%, with a mean value of 3.27% (SD = 0.75). These results quantitatively confirm the graphical comparison presented in Figure 9, demonstrating that the filament assembly model consistently achieves smaller deviations from experimental measurements across all weave types and density combinations. The statistical metrics therefore serve only to reinforce the observed improvement in predictive accuracy associated with filament-level geometric representation.

### 3.4. Discussion

The present study advances the structure–property understanding of woven textile materials by demonstrating that air permeability prediction accuracy is governed by filament-level geometric fidelity. While previous modeling approaches frequently rely on simplified yarn centerline representations, the results presented here (Table 2 and Table 3 and Figure 9) quantitatively confirm that resolving filament packing architecture substantially improves predictive performance across all investigated weave types and density configurations. The observed reduction in mean prediction error from 7.14% (single-line yarn model) to 3.27% (filament assembly model) is directly attributable to the explicit representation of intra-yarn pore morphology. In the simplified model, airflow is restricted to inter-yarn gaps, producing artificially concentrated velocity gradients and localized jet-like acceleration. By contrast, the filament assembly model captures multiscale pore continuity arising from filament diameter, layer arrangement, and cross-sectional packing structure. This geometric resolution enables more realistic velocity redistribution within pore constrictions and smoother downstream pressure recovery, resulting in improved agreement with ISO 9237 [41] experimental measurements.

Importantly, the findings reinforce that air permeability in woven materials is not solely a function of bulk porosity, but rather of pore topology, connectivity, and constriction geometry. Increasing warp and weft set density systematically reduces the effective pore diameter, thereby increasing viscous dissipation and lowering the volumetric flow rate. However, weave architecture modulates this effect. Plain weaves, characterized by high interlacement frequency and shorter float segments, exhibit increased flow tortuosity and reduced permeability. Twill and rib structures, providing longer float regions and more continuous pore channels, demonstrate higher permeability under equivalent density conditions. These structural distinctions are consistently captured by the filament-resolved model, confirming its capability to link microstructural configuration to macroscopic transport behavior.

The deterministic nature of the proposed CAD/CFD framework further enhances reproducibility. Permeability predictions are derived directly from measured structural parameters without empirical fitting coefficients or calibration constants. Consequently, the modeling approach maintains physical interpretability while reducing dependence on regression-based approximations. The agreement observed across the six weave patterns and four density combinations supports the general applicability of the method within the investigated structural domain.

It is acknowledged that the present framework assumes idealized yarn geometry derived from averaged structural measurements and does not explicitly incorporate stochastic fiber migration, surface hairiness, or dynamic fluid–structure interaction effects. These factors may influence localized pore resistance under realistic industrial production conditions. However, isolating geometric resolution effects was essential to rigorously establish the governing role of filament-level morphology in airflow transport. Building upon this foundation, future research may extend the framework to incorporate stochastic microstructural variability, deformable yarn modeling, multilayer textile assemblies, and more advanced flow simulations. Such developments will further enhance predictive robustness and expand the applicability of the methodology toward next-generation digital textile engineering systems.

## 4. Conclusions

This study establishes the development and validation of a three-dimensional filament assembly model for predicting the air permeability of woven fabrics composed of spun yarns. By addressing the limitations of conventional single-line yarn representations, the proposed CAD/CFD framework incorporates filament-level geometric resolution—including filament diameter and cross-sectional layering—to construct physically consistent pore topology across multiple weave patterns and set densities. Validation against standardized experimental air permeability measurements confirms that the filament assembly model consistently achieves high predictive accuracy, with substantially lower percentage prediction errors than the single-line yarn path model. These quantitative results demonstrate the robustness and reliability of filament-resolved geometric modeling under the investigated conditions. The findings further establish a direct micro-scale to macro-scale relationship, revealing that airflow resistance and permeability behavior emerge from filament-level pore morphology and multiscale structural interactions rather than from simplified bulk porosity assumptions. By resolving both intra-yarn and inter-yarn airflow pathways, the model captures realistic flow redistribution mechanisms that govern fabric breathability. From an engineering perspective, the integrated CAD/CFD methodology provides a scalable virtual prototyping platform for performance-driven textile design, enabling accurate permeability prediction and structural optimization prior to manufacturing.

## Figures and Tables

**Table 1:** Set and construction parameters for 24 woven fabric samples [9].

Sample	Type of Weave	Set Density (Warp/Weft)	Mass (g/m^2^)	Thickness (mm)
1	Plain weave	22/15	143.91	0.439
2	(1 × 1 PL)	22/20	166.59	0.438
3		29.3/15	180.41	0.468
4		29.3/20	209.50	0.514
5	Basket weave	22/15	140.52	0.506
6	(2 × 2 BW)	22/20	160.90	0.531
7		29.3/15	173.12	0.604
8		29.3/20	195.98	0.567
9	Twill 3 × 1	22/15	141.36	0.565
10	(1 × 3 T)	22/20	162.24	0.558
11		29.3/15	176.61	0.596
12		29.3/20	198.11	0.586
13	Twill 2 × 2	22/15	141.78	0.508
14	(2 × 2 T)	22/20	160.66	0.504
15		29.3/15	173.34	0.604
16		29.3/20	197.48	0.568
17	Filling rib 4 × 2	22/15	138.14	0.591
18	(4 × 2 R)	22/20	160.60	0.558
19		29.3/15	171.53	0.620
20		29.3/20	196.30	0.605
21	Warp rib 2 × 4	22/15	143.77	0.549
22	(2 × 4 R)	22/20	163.89	0.557
23		29.3/15	179.93	0.471
24		29.3/20	202.52	0.480

**Table 2:** Numerical and experimental results of air permeability.

Type of Weave	Set Density (Warp/Weft)	Experimental Results (L/m^2^/s) [9]	Prediction Results (L/m^2^/s)		% Error	
			Single Line of Yarn	Filament Assembly Model	Single Line of Yarn	Filament Assembly Model
1 × 1 Plain weave (1 × 1 PL)	22/15	2391.67	2551.8	2482.2	6.69	3.78
	22/20	1571.67	1693.4	1655.8	7.74	5.35
	29.3/15	1268.33	1349.4	1308.1	6.39	3.13
	29.3/20	469.67	502.8	482.2	7.05	2.66
2 × 2 Basket Weave (2 × 2 BW)	22/15	3065.00	3297.1	3165.7	7.57	3.29
	22/20	2186.67	2319.9	2246.7	6.09	2.75
	29.3/15	2019.17	2179.1	2082.9	7.92	3.16
	29.3/20	1209.17	1289.5	1256.3	6.64	3.90
1 × 3 Twill (1 × 3 T)	22/15	3505.83	3782.9	3632.5	7.90	3.61
	22/20	2161.67	2319.6	2242.2	7.31	3.73
	29.3/15	1579.17	1679.4	1642.7	6.35	4.02
	29.3/20	833.00	898.6	861.3	7.88	3.40
2 × 2 Twill (2 × 2 T)	22/15	3016.25	3249.0	3130.0	7.72	3.77
	22/20	2023.33	2183.4	2076.6	7.91	2.63
	29.3/15	1681.67	1787.1	1722.9	6.27	2.45
	29.3/20	885.50	951.5	903.5	7.45	2.03
4 × 2 Filling rib (4 × 2 R)	22/15	3491.67	3765.5	3627.0	7.84	3.88
	22/20	2352.50	2531.3	2416.8	7.60	2.73
	29.3/15	2241.67	2379.8	2295.8	6.16	2.41
	29.3/20	1325.00	1428.5	1363.5	7.81	2.91
2 × 4 Warp rib (2 × 4 R)	22/15	3288.33	3522.7	3367.8	7.13	2.42
	22/20	2247.50	2391.0	2341.0	6.38	4.16
	29.3/15	1395.00	1495.8	1445.8	7.23	3.64
	29.3/20	898.33	955.2	922.2	6.33	2.66

**Table 3:** The performance of the prediction models against experimental air permeability data.

Model	% Error			
	Min	Max	Mean	SD
Single line of yarn model	6.09	7.92	7.14	0.67
Filament assembly model	2.03	5.35	3.27	0.75

## Data Availability

The original contributions presented in this study are included in the article. Further inquiries can be directed to the corresponding author.
